# White Collars, Dark Histories: The Factors That Lead Women to Commit Corporate Crimes

**DOI:** 10.1177/0306624X221124837

**Published:** 2022-09-29

**Authors:** Tomer Einat, Lilach Ben-Moshe

**Affiliations:** 1 Bar-Ilan University Faculty of Social Sciences, Ramat-Gan, Israel

**Keywords:** problematic family background, women, love, white-collar crime, ex-prisoners

## Abstract

The literature on white-collar crimes committed by women is sparse, dealing mostly with their motivations, the incidence of the phenomenon, and differences between the women who commit them and those who commit other types of offenses. This qualitative study maps factors leading women to commit such crimes, with particular focus on their family and personal histories, and on the various roles they played as children and adults, which prepared and “trained” them for future illegal behavior. Data were collected using semi-structured interviews with 18 women convicted and imprisoned for white-collar crimes. A relationship was found between problematic family background and difficulty in help seeking and a nearly obsessive need for love in adulthood, and between the latter and white-collar crime. Theoretical and practical conclusions are discussed and future directions proposed.

## Introduction

We appear to know very little about white-collar criminality by women, as most of the literature is about men (Williams & Milton, 2015) and by men (Chesney-Lind & Pasko, 2004). This almost exclusively quantitative literature focuses on four main content areas. First, the definition of the phenomenon: the term was first coined by Sutherland (1949) and was subsequently defined in greater detail (Croall, 2009; Friedrichs, 2004; van Gelder & de Vries, 2016). Second, the offenders’ demographic characteristics (Duffield & Grabosky, 2001; Jordanoska & Schoultz, 2019; Orbach & Huang, 2018; Williams & Milton, 2015), and the differences between them and offenders in other criminal areas (Simpson, 2019). Third, the factors and motivations underlying white-collar crime (Benson & Cullen, 2017; Coleman, 1987; Kawasaki, 2019). For example, Boman and Freng (2017) identified eight types of factors that lead individuals to commit such crimes, and Braithwaite addressed to three major factors that increase the likelihood of involvement in white-collar crime in the organizational context. Finally, researchers have addressed the scope of the phenomenon (May & Payne, 2018).

The few studies about women white collar criminals have concentrated on three main topics. The first is frequency: various researchers have pointed to the lower rate of women who commit white-collar crimes, compared to men, mainly due to fewer opportunities for such crimes in workplaces (Benson & Gottschalk, 2015; Wells, 2019). The second topic is, again, factors and motivations, with various researchers pointing to a link between moral judgment (Gilligan, 1982) and social and/or financial stressors (Goldstraw et al., 2005) on the one hand, and white-collar crime on the other. Finally, researchers have examined demographic and sociodemographic differences between white-collar and other types of criminality (Benson & Chio, 2019; Severson et al., 2019; Shechory et al., 2011).

With regard to *frequency*, Wells (2019) compared American women and men and found that women were significantly less prone to white-collar criminality, arguably due to lack of opportunities open to high-ranking executives ). Others reported similar findings (Benson & Gottschalk, 2015; Steffensmeier & Allan, 2000; Steffensmeier et al., 2013). These studies contradict the basic assumption of opportunity theory (Dodge, 2019) that the massive integration of women in the workplace over the past decades (Benson & Cullen, 2018 ), the growing number of women in top positions (Braithwaite, 2020), their increased access to financial resources and crime opportunities (Campaniello & Gavrilova, 2018), and their lower likelihood of being caught (Felson & Cohen, 2017 ; Kawasaki, 2019), would increase their involvement in white collar criminality, particularly small-scale fraud (Goldstraw et al., 2005; Mishra et al., 2017).

Gottschalk and Smith (2015) found that in 2009 to 2011, only eight out of the 179 offenses covered by three Norwegian newspapers were committed by women. The researchers attributed this low coverage of women’s white collar criminality to a capitalist ideology and the stereotype that businesswomen are less appreciated and less successful than businessmen, who are seen as cultural heroes, as well as to the enduring chivalry model, according to which women criminals in general are either victims or demons (van San, 2011). A subsequent study by Gottschalk (2019) examined views of business administration students on women’s involvement in white-collar crimes. Again, women’s involvement in white-collar crime was underestimated, in line with findings regarding their involvement in other types of crime (Walters & Morgan, 2019 ).

Regarding the *unique characteristics* of female white-collar criminals, Shechory et al. (2011) found that they were usually professionals, and older, more intelligent and educated compared to women who committed violent or drug offenses. Compared to the latter, the former also had few behavioral problems in childhood, began offending at a later age and had little to no prior history, they served in the military, were better educated, and were not exposed to drug or alcohol use in their nuclear family (for similar findings, see Benson & Chio, 2019, and Severson et al., 2019).

With regard to *motivations*, Gilligan (1982) found that for women criminals (of all types), moral judgment was often based on an ethics of care, leading them to try to solve other people’s problems in various ways, including illegal ones. More recently, Goldstraw et al. (2005) examined 155 women responsible for large-scale fraud (over $100,000). They identified five main motives: (1) Pressure by another (usually a male partner); (2) Greed; (3) Gambling debts; (4) Personal and business financial stress; and (5) Attempt to save a struggling business. The researchers concluded that compared to men for whom the fifth motive was dominant, women were motivated mainly by interpersonal relations (for similar findings, see Benson & Harbinson, 2020; Klenowski et al., 2011).

The sparse literature on the factors and motives leading women to commit white-collar crime (Chesney-Lind & Pasko, 2004; Gondles, 2001) is surprising, giving the plethora of studies focusing on men who committed corporate crimes or women who committed other types of crime (Agozino, 2018; Chesney-Lind & Pasko, 2004; Kruttschnitt, 2013; Taylor, 2015). We therefore seek to provide insight into the inner world of female white-collar offenders, their characteristics and motivations, thus narrowing some of the theoretical and empirical gaps in the field. Accordingly, the present study examines and analyzes the relationship between women white-collar criminals’ personal, family and social histories and their criminal conduct in maturity, based on their own subjective, introspective and retroactive accounts.

## Method

This study is conducted in the spirit of qualitative research (Creswell, 2007), informed by the critical interpretive paradigm (Gergen, 2009) according to which various phenomena and problems in the world should be examined in terms of the meanings associated with them by those who experience them (Charmaz, 2014; Denzin, 2017; Ryan, 2006). Accordingly, we do not intend to answer any question derived from a preliminary hypothesis or to maintain a consistent structure, but rather to remain flexible in line with the findings as they are revealed. This flexibility enables access to important yet unpredictable issues that would not have been revealed using a different approach. It also helps determine the relevance of certain emerging issues with regard to the overall topic (Silverman, 2013). Specifically, it enables us to analyze the relations between women’s family and social histories and their motivations to commit white-collar crimes as adults.

## Participants

Eighteen female prisoners on parole convicted of white-collar crimes (i.e., fraud and fiscal offenses) participated in the study, following prison terms of 9 months to 15 years. These women represented about 63% of all women prisoners convicted of such crimes in Israel in 2017–21. They were released from custody up to 2 years prior to the study. Their ages ranged from 30 to 69 years (*M* = 57); twelve were divorced, five single and one a widow. Fifteen were mothers. None had a prior criminal record. Five had high-school education, seven had postsecondary education and six were academics.

## Data Collection

Our data were collected using in-depth semi-structured interviews (Hugh-Jones, 2010), which provides rich, relevant and reliable information (Fontana & Frey, 2005) while allowing for sensitive attention to the participants’ lived experience (Silverman, 2006). The study was conducted in three regional units of Prisoner Rehabilitation Authority (PRA) in Israel. After obtaining ethical approvals from the PRA and the Ethics Committee of the Criminology Department at Bar-Ilan University, one of the authors met the unit directors to explain about the study and ask their help in locating potential participants on a voluntary basis. The researchers contacted these candidates on the phone, described the study objectives and explained that the interviews would be audiotaped and transcribed. After obtaining their verbal consent, the interviews were scheduled according to the participants’ convenience.

Prior to the interview, it was explained to the participants that non-participation or the cessation of the interview at any point would not affect them in any way, that their anonymity would be protected using pseudonyms, and that their narratives would remain confidential. Next, each interviewee signed an informed consent form. The face-to-face interview (approximately 120 minutes in duration) began with demographic questions, followed by open questions designed to elicit the participants’ narratives. Prior to and during the interview, the participants were encouraged to contact therapy professionals in the units for support.

Although 22 women prisoners on parole who had been convicted of white-collar crimes volunteered to take part in the study (approximately 63% of all women prisoners convicted of such crimes in Israel in 2017–21), qualitative data collection reached code and meaning saturation at 18 interviews, when it was noted that the range of common thematic issues identified had become stable and no additional insight emerged. According to Hennink et al. (2017), code saturation is typically reached after nine interviews and meaning saturation between 6 and 24. In addition, the interviews indicated that the dynamics that had led the participants to commit white-collar crimes (e.g., family and dyadic relations or difficulties in seeking help) were highly similar. Hence, upon completing these 18 interviews, we assumed that we had reached theoretical saturation as well.

## Data Analysis

The interviews were thematically analyzed (Chandra & Shang, 2019) with the aim of understanding how the participants subjectively perceived the factors and motivations that had led them to commit white-collar crimes. Following Carey (2017) and Lakshmi and Mohideen (2013), the analysis proceeded in three stages. First, key relevant themes emerging from the interview transcripts were identified (thematic relevance). Next, participants’ interpretations of the link between their family backgrounds and their ways of thinking and behaving in family and society as adults were identified (interpretive relevance). Finally, participants’ motivations for committing white-collar crimes were analyzed (motivational relevance).

## Validity

To ensure the validity and credibility of the findings, two methods were implemented (Golafshani, 2003). First, we strictly followed a uniform interview protocol and provided a complete and accurate presentation of the interviews’ contents. All the interview transcripts were analyzed separately and autonomously by the two researchers to avoid bias (Miles et al., 2013). Any findings or interpretations that they deemed biased or about which they had conflicting opinions were subsequently discussed until they reached a consensual interpretation (Tracy, 2010). Second, the instruments used to conduct the study and analyze the findings were uniform throughout (“dependability”; Gasson, 2004), and matched those used in other studies in the same content area. Moreover, we took extreme care to monitor and document the activities required to collect and analyze our data (Morrow, 2005).

## Results

Four content categories arose from the analysis, and are presented below according to the order in which the participants discussed them: (1) Difficult family background as “preparation” for criminality; (2) Ways of coping with that background and their future implications; (3) Difficulty seeking help; and (4) The circumstances and causes of the crime.

### (1) Family Background as Groundwork for Antisocial Behavior

Many studies have found relations between problematic family background and emotional difficulties among children and the subsequent development of antisocial or criminal behavior (Mumford et al., 2019; Tossone et al., 2018). Aligned with those findings and with particular focus on their relations with their parents, all participants pointed at two major family problems: poor communication and lies. *Poor communication* was manifested in the parents’ indifference or even complete disregard to them:

Since childhood, nobody at home really knew anything about the others. No questions asked, no interest expressed (Hagit, 61, divorced + 4 children).

[Like me,] some of the women who offend are battered women; not battered violently, battered in their soul. It’s more difficult. If I were beaten physically, maybe I’d get up and go [. . .] but the beatings were invisible [. . .] you couldn’t tell anyone because it was at home [. . .]. Mentally, I’ve never felt like a human being (Moran, 44, divorced +2 children).

This disregard involved avoidance of physical and verbal expressions of love:

We never had warmth – never, never, never. [. . .] Only when my mom was dying, she’d hold my hand and say she loved me (Ronit, 49, divorced +3 children).

I envied kids who were hospitalized, you dig? I wanted to be like them – they have love, affection, attention [. . .] they’re hugged, lots of kisses (Galit, 58, single +4 children).

All participants described frequent *lying* in their interactions with their parents, and between their parents and the extended family and community:

You feel there are [. . .] lies as well as secrets you must keep and lie to others. This was with me since as long as I can remember (Tzufit, 53, divorced +3 children).

My dad was a gambler. One night we have a home and the next day he shows up with cardboard boxes saying we’re moving. People always thought our dad was a millionaire. But we knew he wasn’t and that this was a lie, and never spoke about it outside the house, not even in the extended family (Yasmin, divorced +2 children).

This family atmosphere led the participants to adopt a deceptive and antisocial behavior (in addition to verbal lying):

Things that happened on the way foreshadowed the future – gray stuff. For example, my father would hang his trousers in the bathroom and each of the kid took money from the pocket without saying (Dorit, 51, single).

When I was little I would take and do stuff nobody knew about. For example, my father had a coin collection and I would take from it and buy candy. To such a point that they called my mom from the store to ask where I got the money (Dana, divorced +2 children).

Exacerbated due to the parents’ indifference, this situation culminated in complete confusion regarding moral boundaries, preparing the ground for future criminality

I had no problem using my father’s credit card without his knowledge, and then asking for his forgiveness when he found out. [. . .] he didn’t do anything. Perhaps if he responded to it, if he stopped it, saying it was stealing, then it would have made me behave differently in the future (Adi, 52, separated +2 children).

I remember my mother telling me – wait ‘till daddy comes, what he’ll do to you. I was afraid [. . .] and nothing happened. She was just bluffing. She didn’t tell him at all [. . .]. So I kept doing it. . . (Galit).

### (2) Ways of Coping and Their Future Implications

The participants’ main way of coping with their difficult family reality, as both children and adults, was to take control and responsibility for the household. *In childhood*, this was manifested in catering to their family members’ instrumental (instrumental parentification; Kim & Tasker, 2013) and emotional needs (expressive parentification, Perrin et al., 2013). Two participants had this to say with regard to the instrumental aspect:

I gave the orders, what needed to be done. How to pay and whom. At a young age, really. I was angry if they [the parents] didn’t [. . .] have money, so I would give them money from my salary. I’d by them food (Sigalit, 60, divorced +2 children).

I was the problem solver – they had their water supply disconnected because they didn’t have money [. . .], I was the one who went to the municipality, already as a teenager, and I [. . .] reached all the way to the deputy mayor (Tikva, 54, separated +2 children).

In terms of expressiveness, the women demonstrated empathy and support for their families, catering to their mental needs:

I was the problem solver [. . .]. As a young girl I was already working and taking care of everything. They’d come to me with every problem. Every time anyone of them was sad or stressed out or angry, they’d come to me (Anat, 30, in a relationship +2 children).

Everything, really. My dad would come to seek my advice or help. When he was sad, when he was mad at my mom [. . .]. They were like two kids, they don’t have basic parenting skills (Lilach, separated +3 children).

*In adulthood*, the participants became dominant if not domineering in their partners’ lives, managed the household autonomously and showed close involvement in raising their children, giving them the love and warmth they lacked as children. With regard to their relationships, all emphasized that they knowingly chose a weak and passive male partner in order to feel strong and meaningful (see Schoppe-Sullivan et al., 2015).

My partner choices are part of the problem. It was a need for control [. . .]. I chose men who needed help. It was part of this thing of doing whatever I wanted and by myself, for good and for bad (Adi).

I always chose whoever was willing to accept that the final, strong word would be mine [. . .] (Tzufit).

In managing the household and family, the participants had exclusive control. Anat “did not let him intervene. [. . .] We both knew our parts and got along splendidly.” Sigalit was “in charge of everything.”

Finally, the participants admitted to giving their children generous and often excessive doses of love. Adi “tried to be a very [. . .] warm mother for my children, so that they’ll say I’m good at least in that. And so it was, I was good at it!” Similarly, Tzufit boasted: “One thing I was good at was raising my children. I gave it all [. . .] I was totally there for them!” Note that this attitude was motivated not only by caring for the children, but also out of the participants’ need to compensate for their own childhood deficiencies and experience meaning and success in life. Anat and Hagit (respectively) described this explicitly:

I don’t want my children to have what I had. I tell them I love them ten times a day, hug them every day [. . .] so that they know there’s a mom here, something I didn’t have. I’d never wake my kids up to clean up the house. [. . .] No way they’d miss a school trip because there’s no money, inconceivable. [. . .] I won’t have them feel invisible, unloved, having to do what a mother has to do.

I had nothing, that’s why I wanted to give my children. So that they’d love me. [. . .] I wanted to give in order to be loved.

The participants employed this parenting style, in both its instrumental and emotional aspects, both prior to and while offending. Thus, in a kind of double life, they went on committing crime while functioning as mothers—optimally, from their perspective:

I was a volunteer at school, a well-known figure, chair of the parents’ committee; they loved me and wanted to be next to me. I give my all. And on the other hand, I do forbidden things at the same time [. . .] I’m a lousy thief! (Adi)

As a mother I did everything I had to but I was also hunted [. . .]. The kids felt there was really a mom going out to the park with them [. . .] and all that when I have debts and I’m hunted and I’m busy finding practical solutions for my troubles (Tzufit).

Even after their imprisonment, the women remained dominant in their children’s lives, and maintained continuous and positive relations with them (at least in their view), mainly through multiple daily phone calls in which they made decisions with and for them:

One of the most difficult things about prison was the distance from my kids. But we kept talking on the phone [. . .]. And I also decided everything – who would teach them, when to buy things for them (Tikva).

We found ourselves talking for hours on the phone, about everything. Every decision, every choice [. . .]. We thought about everything together. [. . .] and then when I would go out on leaves [. . .]. It strengthened our relationship very much (Hagit).

### (3) Difficulty Seeking Help

Various studies associate avoidance of help seeking with fear of damage to one’s self-image and sense of efficacy (Prioste et al., 2015; Zhao et al., 2015). Consistent with and supporting these findings, many participants described having avoided help seeking as children out of fear for their self- and social esteem, consciously preferring to cope independently. Rachel (52, divorced +2 children), for example, had a “terrible time asking for and receiving help,” and admitted this was still true. Galit felt that if she told anyone she was having trouble and did not know what to do “they’ll think I’m no good [. . .]. The desire to show others that everything’s fine with me and this way to think I’m really strong was always there.”

This avoidance was often supported by educational messages stressing they had to be independent and strong. Rachel was raised “Never to ask help from anyone,” and Ronni (59, divorced +3 children) was taught that she could always cope, “So how can I say I can’t? It’s like I’m weak!”

As suggested, the participants kept avoiding help seeking as adults, including in the financial area, and tried coping independently even when this led to further complications:

When there was a financial problem, I thought it was a shame to ask for help [. . .]. I thought you had to please the entire world and to whatever it takes. I paid a huge price for that (Bracha, 30, divorced with no children).

At that time I didn’t know how to ask for help. I couldn’t tell my husband – listen, someone’s blackmailing me. I blame only myself for that [. . .]. I took the [high interest] loan. I could’ve asked for help. Nobody knew [. . .]. Today I know that when I grew up in this survival mode [. . .] I learned to manage all by myself (Tzufit).

Note that the persistent avoidance of help seeking in both childhood and adulthood and the insistence of coping independently found in this study are contradictory to the consensus in the literature that both girls and women tended to seek help rather frequently (Williams, 2014), mainly due to their gender role socialization (Hance et al., 2018).

### (4) Circumstances and Causes of the Crime

#### Money

According to the participants, the only immediate and concrete reason leading them to break the law was financial:

I owed money in my business. Not to people, to banks. [. . .] I had to make at least 30,000 NIS [$10,000] every day, and in the business, some days you have work and some days you don’t. So I entered the loan shark market, I took from the one and gave the other [. . .] (Rivka, 69, widow +2 children).

I needed the money [. . .] to pay so that my kids would survive. Several months before I was arrested, I decided to sell my kidney because there was no longer anyone I could take from. . . (Sigalit, 60, divorced +2 children).

However, deeper analysis of the transcripts reveals two additional and complementary causal factors: need for love and the criminal spiral.

#### Need for love

In the criminological context, love has been associated with avoidance or desistance from antisocial or criminal behaviors (Forste et al., 2011). Lack of love expressions by one’s social environment often leads to a permanent sense of anger and twisted moral worldviews (Yeo & Chan, 2020), related to criminal behavior in adolescence and adulthood (Fraser, 2004; Gawda, 2012).

Consistently with the literature, many participants associated lack of love in their childhood and an immanent and dramatic need or desire to feel it as adults with their criminal activities. Adi described herself as a kind of “Robbin Hood – the good fairy who will sort everything out for everyone except herself. Do good to others, and they’ll love you.” Bracha expressed a similar lifelong need “to be loved, to be seen, to feel significant, that they can’t do without me. For that, I was willing to get into trouble again and again.”

These participants realized this emotional deprivation-anti-sociality-criminality nexus, both while offending and at an earlier age. Anat had “a very unhealthy” and “mutually abusive” relationship with her brother, which “made me feel important and loved by someone. That’s when the lies and secrets began – at age 10!” Ronit confessed to starting lying at a young age, “To make them love you and say good things to you and praise you. [. . .] It kept on like that and became more sophisticated with the years.”

#### Criminal spin

Ronel’s (2011) Criminal Spin Model explains how individuals become carried away into criminality from their own perspective. According to this model, the individual begins by underestimating the risks and damages of offending, and then gradually loses control until it becomes almost impossible to stop (Zemel et al., 2018). Indeed, Adi described it as an “addiction – difficulty to delay gratification. [. . .] when you do it once, it’s very easy to go down that road again.” According to Yasmin, a financial fraudster “is like a junkie, constantly thinking how to pull another scam.”

Other participants focused on the selfishness aspect of the Criminal Spin Model (Ronel, 2009, 2011). Their existential and nearly uncontrollable need to help others through criminal activity served, albeit somewhat paradoxically, their egotist fantasy of feeling important and acting like “saviors,” since this activity expressed their love for others and enabled them to receive love in return. Thus, trapped in a vicious cycle, whenever their “emotional hole” became empty, they chose to help others by offending in return for love and warmth:

I felt like superman. I supported my brother [. . .] met the expectations. [. . .] and he showed me that he appreciated it [. . .]. It made me feel good, so I went on [. . .] (Adi).

I wanted my dad to tell me, good job, I’m proud of you my beloved daughter. To hear him say that, I began and went on stealing [. . .]. I’d do everything to hear that word! (Bracha).

This constantly accelerating spin gradually led to total loss of control over the criminal behavior, which became part of the participants’ lives: “the power of darkness really took over me,” said Tzufit. For Adi it was about “lies that became part of life. [. . .] every day I promised that this was the last one, and then I went on.”

Nevertheless, at some point, the women became fatigued, despaired and in some cases also starting losing their mind:

I told myself – I used to steal, I know how to lie. I stole once when I was a kid, I remember how to do it. [. . .] You know, I wear a mask. And then you have lots of characters inside you. But then I got tired of that (Anat).

I felt I was losing my mind. I went to a psychiatrist for a year. [. . .] He said I didn’t have a mental illness; that I created my illness, maybe wanted attention I hadn’t gotten [. . .]. I wanted to scream for all to hear – I’m in deep trouble for years! (Ronni)

I tried to commit suicide several times. I took Clonex [Clonazepam] for a long time because I an anxious and I felt I couldn’t go on anymore (Moran, 44, divorced +2 children).

I tried to kill myself twice before everything was discovered, and again three months after that. My daughter, who lives with me, started going to bed with me because she was afraid I’d do something to myself (Hagit).

## Discussion

Multiple studies have examined the relations between women’s life circumstances, family and social history and victimhood and their criminal behavior (e.g., Kruttschnitt, 2013; Taylor, 2015). Nevertheless, to the best of our knowledge this has not yet been done in the white-collar crime area. The present qualitative-interpretive study examines these relations based on the retrospective introspections of 18 Israeli convicted criminals.

The first main finding addressed the women’s problematic family background, as it relates to their criminality. Similarly, several studies have found a relation between lack of support, warmth and love in childhood and multiple mental issues in adulthood (Pasco Fearon & Roisman, 2017; Schimmenti et al., 2015; Sternberg, 2006). These studies, as well as others (Boboc, 2017) also found that children lacking in family love and warmth tend to seek them elsewhere, which represents a risk factor for the future development of antisocial behavior, risk-taking tendencies, low self-esteem and antisocial personality (Farrington, 2020; Yeo & Chan, 2020). Fraser (2004) associated this background specifically with antisocial and criminal behavior among girls.

All participants suffered from emotional neglect as children, leading them to do whatever they could to experience appreciation and love elsewhere. At first, they did it in legitimate ways, albeit at a high personal cost, by taking responsibility for providing emotional support for their family members. Subsequently, given the powerful expressions of love they received in return to their illegal help, they adopted criminal behaviors.

Other studies found a relation between lack of parental attention and children’s tendency to lie, to be dependent, and to have difficulties with emotional regulation, relations and self-esteem as adults (Luyten & Blatt, 2013; Pepin & Banyard, 2006). All participants testified that their parents’ lying and tendency to ignore their lies as children led to moral confusion, and resulted in the persistence of that behavior into adulthood. Derksen (2012) argued that the main motivation of the ordinary person for lying, particularly in situations of uncertainty and danger, was controlling others. Indeed, it appears that for the participants, lying was a way of controlling their family, but at the same time it made them feel defensive and that they were living in a survival mode, potentially paving the way for similar, criminal behaviors in the future. Lying and concealing became a permanent feature in their lives.

Our second main finding is that white-collar criminals have coped as children mainly through parentification (Perrin et al., 2013). The gap between the mental maturity required for running the household and their chronological age led them to conceal their own emotional distress and develop a twisted behavioral pattern whereby their only way to feel loved was to almost completely renounce their own needs. They led a double life: communicating strength and maturity on the outside, but feeling weak and insecure on the inside.

As a result, many of the women experienced emptiness, loneliness and low self-esteem as adults, adopting pathological behavioral patterns whereby whatever they did had to gain others’ approval and appreciation (see Kim & Tasker, 2013).

To the best of our understanding, these patterns only exacerbated their difficulty, leading them to take control over their loved ones, which made them feel powerful and meaningful, at least temporarily (Bargai et al., 2007), and contributed to their wellbeing. Their control over their partner—conceptualized in the literature as maternal gatekeeping (Schoppe-Sullivan & Altenburger, 2019)—empowered them and made them feel responsible for all aspects of the family and household, enabling them to commit their crimes with relative ease, without their passive and weak partners even noticing their problem behaviors.

The women were also dominant in raising their children. According to their narratives, they seem adopted a democratic parenting style (Campbell & Gilmore, 2007)—diametrically opposed to their own authoritative education. Placing the children at the center was rewarding for them (see Levendosky et al., 2006; Peled & Dekel, 2010), and contributed to their self-perception as successful mothers (Bargai et al., 2007).

The third finding relates to the women’s difficulty to seek help. Bowlby (1988) argued that an individual’s motivation to seek help in distress depended on their assessment of their internal and external resources. Thus, an individual who has experienced inconsistent parental response as a child would adopt ineffective problem-solving strategies as an adult, find it difficult to regulate emotions, and avoid seeking help from significant others (Vogel et al., 2005; Wu et al., 2010). Bowlby (1988) also addressed the psychological price the help seeker needed to pay: asking for help may reduce distress and improve social interactions with others, but may also entail embarrassment, weakness, a sense of dependency and inferiority, and an overall threat to one’s self-esteem (van Heeringen, 2001).

In keeping with these explanations, the participants avoided seeking help throughout their lives, having realized at a young age that their environment ignored them and that any request would be rejected. As a result they became extremely self-reliant, feeling that they could solve their problems alone, preferring to give up on the potential instrumental benefits of help seeking in order to avoid the psychologically disempowering price it entailed.

Finally, the findings highlighted how the women were carried away into criminal activity and lost control (Zemel et al., 2018). Ronel’s (2011) Criminal Spin Model describes how criminals become increasingly drawn or “addicted” to their criminal behaviors, as some participants described it, unable to stop the escalation of their illegal conduct (Ward et al., 2015).

In line with this model, the participants described a sequence of criminal events that at first was accompanied by pleasure, leading to powerful feelings of control and the necessity to continue. It also made them more confident in their ability to solve their own and their loved ones’ problems independently, feeding back into their criminal behavior, and so on. They also felt exposed to the threat of losing their importance in the eyes of their loved ones and losing their love, with criminality being absolutely *necessary* to remove that threat.

Next to the concrete and emotional profits from their criminality, however, their sense of omnipotence began to shatter. Consistently, they became exhausted of their double lives, losing their minds, and wishing they were dead (see Maruna & Roy, 2007).

In conclusion, the findings of this study suggest a (perceived) relationship between difficult life events in childhood and adolescence and white-collar criminality of adult women. This relationship between emotional victimization of women and their turning to financial crimes has not been identified in the literature, and contradicts the literature on white-collar crime among men, that stresses the role of instrumental environmental-occupational factors (Benson & Gottschalk, 2015; Klenowski et al., 2011).

Our findings contribute to a more nuanced understanding of white-collar crime, and also have therapeutic implications. Since many female white-collar criminals have been subjected to neglect and abuse as children, their treatment must address those experiences. We believe these women need a dignifying therapeutic relationship that will encourage them to share their narrative and take responsibility for their lives, enabling them to remove the mask of the strong and perfect woman and give room to the needy child within them. Accordingly, we recommend that these women be treated in an approach appropriate for people with complex PTSD as part of trauma-informed care (Ford & Blaustein, 2013).

## Limitations and Future Directions

There are three main limitation to this study. The first limitation concerns data validity. Qualitative semi-structured interviews have been used to collect the data. Several studies have indicated that this method can result in under- or over-reporting, affecting the credibility of the interviewees’ testimonies (Krohn et al., 2013). It is therefore possible that using other data collection techniques in addition to interviews, such as ethnographic observation and written self-reports could have produced additional or complementary data, augmenting the validity of our findings.

Another limitation concerns gender bias: although our objective was to examine causes and circumstance leading women to white-collar crime, we cannot ignore the need to compare them to men. Future studies should compare men and women who committed corporate crimes. Such a comparative analysis will allow for a broader and deeper overview of the white-collar crime phenomenon, its unique causes and the role of gender in understanding it (Creswell, 2007).

Finally, a third limitation relates to relatively small sample size: only 18 women convicted of white-collar crimes. Whereas, as mentioned, this has proved sufficient to achieve saturation in the present research design, it does mean that the generalizability of these findings to the population of white-collar women criminals is Israel and globally is limited. Given this limitation, and the exploratory nature of the current study, we believe future studies should use a larger sample and include, for example, an examination of the attitudes of these women’s partners, ex-partners and children toward their relationships with their spouses and mothers, respectively.
